# Search efforts and face recognition: the role of expectations of encounter and within-person variability in prospective person memory

**DOI:** 10.1186/s41235-024-00590-6

**Published:** 2024-09-18

**Authors:** Kara N. Moore, Blake L. Nesmith, Dara U. Zwemer, Chenxin Yu

**Affiliations:** 1 University of Utah, Salt Lake City, USA; 2 Oklahoma State University, Stillwater, USA

**Keywords:** Prospective person memory, Within-person variability, Missing persons, Wanted persons, Attention, Prospective memory

## Abstract

People perform poorly at sighting missing and wanted persons in simulated searches due to attention and face recognition failures. We manipulated participants’ expectations of encountering a target person and the within-person variability of the targets’ photographs studied in a laboratory-based and a field-based prospective person memory task. We hypothesized that within-person variability and expectations of encounter would impact prospective person memory performance, and that expectations would interact with within-person variability to mitigate the effect of variability. Surprisingly, low within-person variability resulted in better performance on the search task than high within-person variability in Experiment one possibly due to the study–test images being rated as more similar in the low variability condition. We found the expected effect of high variability producing more hits for the target whose study–test images were equally similar across variability conditions. There was no effect of variability in Experiment two. Expectations affected performance only in the field-based study (Experiment two), possibly because performance is typically poor in field-based studies. Our research demonstrates some nuance to the effect of within-person variability on search performance and extends existing research demonstrating expectations affect search performance.

## Significance statement

People are unlikely to make a sighting of a missing or wanted person in a simulated search task. Most people do not devote enough attention to searching for the person, because they think it is unlikely that they will encounter the person. Additionally, people perform poorly at recognizing unfamiliar faces, which contributes to people failing to notice a mock missing or wanted person in their midst. Researchers have found that showing searchers photographs of unfamiliar people that showcase the variability in the person’s appearance enhances face recognition and sightings. However, no one has examined how people’s expectations of encountering a mock missing or wanted person impact how helpful seeing the variability in their appearance is to making a sighting. We examined both variables in a computer-based and a real-life simulated search task because people likely have higher expectations of encountering the mock missing or wanted person in the laboratory-based task than the real-life task. Unexpectedly, in the laboratory-based task, participants who saw low variability images performed better than participants who saw high variability images. This may be because the low variability images more closely resembled the missing person’s appearance on the task. In the field-based task, variability did not affect sightings, but expectations of encounter did. Searchers told that there was a high chance of encountering the person performed better than searchers told there was a low chance, even though participants had the same chance to encounter the person immediately after they were asked to search for her.

Authorities use media campaigns to request that the public search for missing and wanted persons (i.e., target persons), but people perform poorly in simulated person searches (Lampinen & Moore, 2016a; Lampinen et al., 2016). People often have low expectations of encountering target persons, which contribute to searchers failing to sight targets in their midst (Lampinen & Moore, 2016a; Moore & Lampinen, 2019; Moore et al., 2016). Additionally, people perform poorly at recognizing unfamiliar faces (Bruce, 1982; Burton & Jenkins, 2011; Hancock et al., 2000; Megreya & Burton, 2008), which leads to searchers failing to sight targets in their midst. Exposure to the variability in a person’s appearance, called within-person variability, improves unfamiliar face learning, or determining whether a face matches the identity of a face represented in one’s memory (Juncu et al., 2020; Menon et al., 2015, Experiments 2–3; Ritchie & Burton, 2017, Experiment 1A; Ritchie et al., 2021, Experiments 2–4; Sweeney & Lampinen, 2012; Experiment 1). Within-person variability improves people’s ability to discern when a target was in their midst on person searches (Juncu et al., 2020; Sweeney & Lampinen, 2012; Experiment 1), but it is unclear how this variable interacts with expectations of encounter. Importantly, low expectations reduce searching for the target person, potentially mitigating any benefit of within-person variability on face recognition.

Person searches are typically studied as prospective memory tasks named prospective person memory, a special case of event-based prospective memory in which participants are asked to report a sighting if they encounter a target person (Lampinen & Moore, 2016b; Lampinen et al., 2008). People perform poorly at prospective person memory, especially outside of the laboratory due, in part, to low expectations of encounter. Participants who learned a target would appear in the building where the study occurred made more sightings than participants who learned the target would appear on campus, even though the target always appeared in the building where the study occurred (Moore et al., 2016). Additionally, participants who encountered targets in an expected context made more sightings than participants who encountered them in an unexpected context (Moore et al., 2018). Participants who searched for one target made more sightings than participants who successively searched for three targets and only encountered the last, suggesting that people’s expectations and attention are affected by failures (Lampinen & Moore, 2016b). Critically, Moore et al. (2016) found that expectations of encounter predicted reported search efforts, which predicted sightings; others have found correlations between expectations and looking behavior (Lampinen et al., 2016). Generally, low expectations of encountering the target reduce attention to searching, which leads to sighting failures (Lampinen & Moore, 2016b; Moore & Lampinen, 2019; Moore et al., 2016).

As for the attention and memory mechanisms needed to make a sighting, noticing the target is a necessity, but noticing a target alone only increased sightings slightly (< 10%) (Moore & Lampinen, 2019). Deliberately searching, called strategic monitoring (McDaniel & Einstein, 2000) or being in retrieval mode (Guynn, 2003), while a target person was in searchers’ midst substantially increased sightings. However, being in retrieval mode did not cause a ceiling effect, potentially because participants had a poor memory of the target’s face.

Unfamiliar face recognition error rates are commonly between 10 and 30% (Burton et al., 1999; Burton & Jenkins, 2011). People perform similarly on face recognition tasks performed after prospective person memory tasks with error rates at around 20–40%, suggesting that face recognition plays a role in searchers failing to make a sighting of the target(s) (Lampinen et al., 2016; Moore et al., 2016; Moore & Lampinen, 2019). Therefore, improving unfamiliar face recognition could improve prospective person memory.

To improve *unfamiliar* face recognition, researchers have built interventions based on theories of why people perform well at *familiar* face recognition (Burton et al., 2016). People are theorized to perform well at recognizing familiar faces because they have built a dynamic representation of the face from viewing it under many conditions (Kramer et al., 2017). On simultaneous unfamiliar face matching, or comparing two images of faces to decide whether they feature the same face, exposing people to the variability in an unfamiliar person’s appearance has improved accuracy on match trials without a concomitant decrease in accuracy on mismatch trials (Mileva & Burton, 2019, Experiments 1–2; Menon et al., 2015, Experiment 1; White et al., 2014). In contrast, some research has found that variability caused an increase in accuracy on match trials at the cost of a decrease in accuracy on mismatch trials on simultaneous face matching (Menon et al., 2015, Experiment 3; Ritchie et al., 2021, Experiments 1 and 2). Additionally, some research finds no effect of exposure to variability on accuracy at simultaneous face matching (Kramer & Reynolds, 2018; Mileva & Burton, 2019, Experiment 3; Ritchie et al., 2020). Exposure to variability has improved accuracy on sequential face matching tasks (Ritchie & Burton, 2017, Experiment 1A). Additionally, some research on sequential face matching has found that variability increased match accuracy without decreasing in mismatch accuracy on sequential face matching tasks (Menon et al., 2015, Experiments 2 and 3; Ritchie et al., 2021, Experiments 2–4). At least one study has found no effect of variability on face learning (Ritchie & Burton, 2017, Experiment 1B). In line with the face matching research finding a benefit to variability, prospective person memory research suggests that variability improves performance without increasing false alarms or response bias (Juncu et al., 2020; Sweeney & Lampinen, Experiment 1).

We posit that exposure to within-person variability may increase familiarity of the studied face, and thus, the automaticity of face recognition given previous research shows that familiar face recognition is more automatic than unfamiliar face recognition (Gobbini et al., 2013; Jung et al., 2013). Increasing automaticity is critical as prospective memory tasks often require people’s limited attentional resources (Einstein & McDaniel, 2005; Moore & Lampinen, 2019; Smith & Bayen, 2004). Evidence for this comes from decrements in response time to ongoing distractor tasks when the prospective memory task is introduced (see Anderson et al., 2019 for review). However, the demands on attention are lower when the cue (e.g., the target person) is salient, the association between the cue and the task (e.g., reporting the sighting) is strong, and the cue and task must rely on the same mechanism (e.g., face recognition) (Einstein et al., 2005). Therefore, high variability photographs may mitigate the impact of low expectations of encounter if they increase the automaticity of recognition and people’s low expectations do not prevent them from searching.

## Current research

We examined the impact of expectations of encounter and within-person variability on prospective person memory in a controlled, laboratory-based paradigm Experiment one and an ecologically valid, field-based paradigm that involved searching for a “missing person” in one’s everyday life Experiment two. Participants saw three high variability (*vs*. low variability) photographs of targets and were told there was a low (20%) or high (90%) chance of encountering the targets. In "Experiment one," we hypothesized that participants who saw high variability photographs would have higher discriminability and be more willing to make sightings (i.e., a more liberal response bias) than participants who saw low variability photographs.^1^ We hypothesized that participants given high expectations of encounter would make more hits and false alarms than participants given low expectations of encounter. We posed two competing hypotheses about the interaction between variability and expectations.^2^ Variability may only affect hits if participants expect to encounter the target and devote effort to searching. If so, variability would only affect sightings in the high expectation condition. In contrast, people with high expectations may not need the benefit of exposure to variability. People with low expectations of encounter may benefit from variability if it makes face recognition automatic. In this case, variability should only have an effect on hits in the low expectation condition.

We hypothesized that participants with high expectations would respond more slowly on the ongoing task, indicating that they were devoting more attention to searching, than participants with low expectations. We formulated two competing hypotheses about the effect of variability on ongoing task response time. First, high variability photographs could improve unfamiliar face recognition, making the prospective person memory task less cognitively demanding. If so, participants in the high variability photograph condition would respond more quickly on the ongoing task as a result of face recognition being less cognitively demanding than participants in the low variability condition. Second, low variability photographs may improve awareness of a person’s typical appearance, allowing participants to form a more consistent mental representation of the target, which would make the prospective person memory task less cognitively demanding. Consequently, participants in the low variability condition might respond more quickly on the ongoing task than those in the high variability condition. Hypotheses for both experiments were registered on OSF prior to data collection (https://osf.io/tymd2/).

## Experiment one

### Method

#### Design

We report how we determined our sample size, all data exclusions (if any), all manipulations, and all measures in the study. Both studies were approved by the ethics committee (IRB-21-362). The study was a 2 (within-person variability: low or high) × 2 (encounter expectations: low—20% or high—90%) between-subjects design. We manipulated variability between-subjects to avoid confounding the variability manipulation because participants studied all targets at once and then searched for them together. The dependent variables were hits (i.e., proportion of accurate sightings of targets), false alarms (i.e., proportion of inaccurate sightings of targets), discriminability, response bias, and attention devoted to searching (i.e., response time on the ongoing task). The study was preregistered on OSF prior to data collection (https://osf.io/fsehj).

#### Participants

A power analysis recommended 351 participants to detect a small effect (*f* = 0.15, *α* = 0.05, 1 − *β* = 0.80) for a between-subjects ANOVA (Faul et al., 2007, 2009). Four hundred and twenty participants were recruited from Prolific. Of them, 68 participants did not complete the study, one participant’s data was lost due to recording errors, and one participant completed the study twice (their second set of data was excluded). Additionally, there were two preregistered exclusion criteria. Participants were excluded if they incorrectly answered two or more of three multiple choice attention and instruction check questions or if they reported being “not at all” or “somewhat” motivated to put effort into their responses in the study. No participants were excluded based on these exclusion criteria. Thus, the final sample consisted of 351 participants. Participants had a mean age of 41.44 (SD = 13.43). Of the participants, 215 (61.25%) identified as male, 127 (36.18%) identified as female, four (1.14%) identified as non-binary, two (0.57%) identified as transgender female, one (0.28%) identified as a transgender male, one (0.28%) identified as gender variant or gender nonconforming, and one (0.28%) preferred not to respond. Additionally, 254 (72.36%) of the participants identified as white, 35 (9.97%) identified as Black or African-American, 30 (8.55%) identified as Hispanic or Latinx, 17 (4.84%) identified as Asian, 11 (3.13%) identified as mixed race, two (0.57%) identified as American Indian or Alaska Native, one (0.28%) identified as a Native Hawaiian or Pacific Islander, and one (0.28%) preferred not to respond.

#### Materials

*Target photographs* Five volunteers provided three sets of ambient photographs of themselves to be used as targets in the prospective person memory task via a Qualtrics survey. The volunteers were recruited by colleagues at other institutions who disseminated the opportunity to students in their classes and members of their laboratories. The survey included a request for at least four low variability photographs of the volunteer taken close in time to one another (i.e., taken within the same day) or that showed the consistency of their facial appearance. The survey also included a request for at least four high variability photographs of the volunteer taken at different times from one another or that showed the variability of their facial appearance over time. The survey included a request for at least two mugshot- or identification document-style photographs of the volunteer that showed the full, frontal view of their face with a neutral expression. The image that best fit the instructions for the mugshot- or identification-style photograph was selected to be used as the test photograph in the prospective person memory task. Each section of the survey provided example photographs for volunteers to use as a reference along with a list of criteria that the photographs needed to meet (see Appendix A). We reviewed each submission and followed up with volunteers if additional photographs were needed. Five volunteers provided photographs and were compensated $20 USD for their time. Four of the volunteers (two women, two men) were selected as targets based on the suitability of their images for the study. For the two targets who were male, target one was white, Hispanic, and 19 years old, and target two was Italian and 20 years old (see Appendix B). For the two targets who were women, target three was white and 21 years old and target four was Hispanic and 19 years old.

*Similarity ratings* Two pilot studies were conducted to classify target photographs into low variability and high variability groups. The classification decisions were made based on the similarity ratings of pairs of photographs of each target. The first pilot study included 30 participants from Prolific who completed a Qualtrics survey that included 48 trials. Each trial included two photographs of one target positioned side by side. Participants were told the photograph pairs were of the same person. Participants rated the similarity in the target’s appearance between the photographs on a 5-point Likert scale (1 = little to no similarities; 5 = almost or exactly the same). We obtained similarity ratings of 12 photograph pairs of each target. Photographs that had an average similarity rating of 3 were replaced with new photographs of the target and were rated by a new group of 30 participants from Prolific. Table 1 shows the average similarity ratings of the target photographs used in "Experiment one." Photograph pairs that had an average rating above 3 were used as low variability study photographs, and photograph pairs that had an average rating below 3 were used as high variability study photographs.

**Table 1:** Mean similarity ratings of the target photographs in "Experiment one"

	Low variability				High variability			
Target number	1	2	3	4	1	2	3	4
*Study images*								
Study image one and two	4.13	4.33	4.47	3.13	2.80	2.63	2.77	2.06
Study image one and three	4.67	4.47	4.07	3.13	2.86	2.63	3.20	2.13
Study image two and three	4.70	4.10	3.50	4.87	3.50	2.07	2.50	2.97
Grand mean	4.50	4.30	4.01	3.77	3.05	2.65	2.82	2.39
*Study and test images*								
Study image one and test	3.87	4.07	3.63	3.16	2.17	3.90	2.63	3.51
Study image two and test	4.37	3.63	3.27	2.35	2.53	2.20	3.93	2.32
Study image three and test	4.47	3.97	4.53	2.51	2.67	2.73	2.33	2.35
Grand Mean	4.24	3.89	3.81	2.67	2.62	2.94	2.96	2.72

**Fig. 1:**
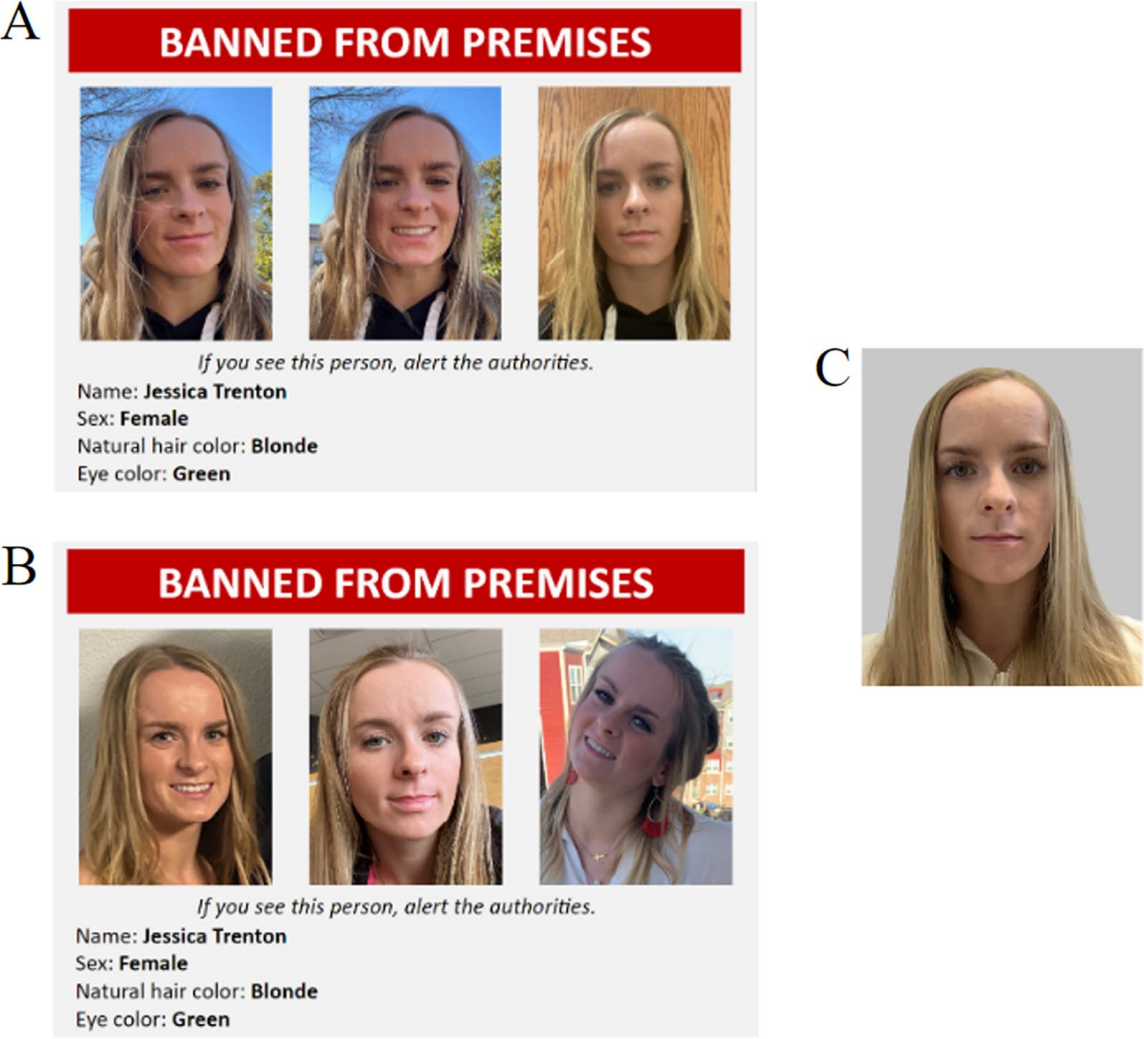
Example banned persons poster of the same target: **A** the low within-person variability version of the banned persons poster, **B** the high within-person variability version of the banned persons poster, **C** the neutral photograph of target shown in the bar-admittance task

*Banned persons posters* We created posters of each target for the study phase of the prospective person memory task. The poster included text that said that the targets were banned from a nightclub’s premises. Two versions of the poster were created for each target: one poster that featured three low variability photographs of the target and one poster that featured three high variability photographs of the target. Each banned person poster included demographic information about the target. Figure 1 shows an example of a banned persons poster.

*Bar-admittance task* A prospective person memory task was created based on Juncu et al.’s (2020) bouncer task. The instructions stated that participants should imagine that they are a bouncer at a nightclub. The instructions outlined that people over the age of 25 years should be permitted to enter (with a press of the ‘Y’ key) and that people under the age of 25 years should be denied entrance (with a press of the ‘N’ key). The bar-admittance task had two blocks of trials. Each trial included a single photograph of an individual that was displayed until a response was made. The first block (i.e., the ongoing only task block) had 46 trials consisting of distractors. The second block (i.e., the bar-admittance task block) had 50 trials with 46 distractors and four “banned” targets. Of the trials, 26 were over 25 years of age and 24 were under 25 years of age (including the 4 targets). Before the second block began, instructions indicated that participants would search for the banned targets and press the ‘A’ key to alert the authorities if they thought they saw one of the banned targets instead of making the age judgment.

*Distractor photographs* We used an equal number of photographs of individuals from the Chicago Face Database (Ma et al., 2015), the Face Research Lab London Set (DeBruine & Jones, 2017), and the FEI database (Thomaz & Giraldi, 2010) to ensure that the target photographs did not systematically vary from the distractor photographs (i.e., that they were forward-facing, portrait-style shots similar to the target photographs). The photographs were split by apparent sex (i.e., female and male) and age (i.e., above or below 25 years old). A pilot study was conducted to norm the perceived age of each attendee photograph. Thirty participants from Prolific rated the perceived age of each person on a 3-point Likert scale (1 = definitely below 25 years of age, 2 = around 25 years of age, and 3 = definitely over 25 years of age). Photographs with a median rating of around 25 years of age were replaced with new individuals whose age was rated by a new group of 30 participants from Prolific to ensure there would be a correct response for the bar-admittance task trials. Photographs of individuals with a median perceived age rating below 25 years of age were categorized as below 25 years old, and photographs of individuals with a median perceived age rating above 25 years of age were categorized as over 25 years old.

*Post-task survey* A post-task survey was created consisting of self-reported questions about the bar-admittance task. The survey began with post-task instruction checks about which keys needed to be pressed to indicate each type of response on the bar-admittance task. The survey included questions about participants' expectations that they would encounter the banned targets on a 4-point Likert scale (1 = not likely at all, 4 = extremely likely), the number of banned targets they thought were displayed on the bar-admittance task block, the number of banned targets they thought they noticed, and a yes/no question about whether participants believed they had a chance to encounter the banned targets.

*Face recognition task* The face recognition task measured the retrospective memory of the banned persons. The task included eight trials with each trial featuring a single photograph of an individual randomly selected from the second block of the bar-admittance task. Half of the photographs were of the banned targets and half of the photographs were of distractors. The instructions said to respond to indicate whether the person was one of the banned persons (press the ‘Y’ key) or not (press the ‘N’ key). The trials advanced when participants made a response.

#### Procedure

After providing informed consent and their Prolific ID via a Qualtrics survey, participants were routed to Millisecond’s Inquisit Web platform (Version 6.6.1) to download a temporary program file to complete the bar-admittance task. First, participants read instructions for the task and then answered two instruction check questions to ensure they understood the task. Participants completed seven practice trials of the ongoing only task and the first block of trials, which were ongoing only task trials. After completing the first block of trials, participants were instructed that they would be on the lookout for four banned persons who may attempt to enter the premises while they assess attendees’ ages. Participants viewed the posters of the four banned persons one at a time for 15 s each and were randomly assigned to see all low or high variability photograph posters. After studying the banned persons posters, participants were informed there was a 20% or 90% chance that the banned persons would attempt to enter the premises. Participants answered instruction check questions to ensure they understood the task before continuing to a filler task. The 3-min filler task consisted of multiple trials in which a sequence of four numbers was shown one at a time, requiring participants to hold the numbers in working memory and sum the total of the sequence. Participants completed the second block (i.e., bar-admittance task trials), the post-task survey, and the face recognition task. Then, participants read a debriefing and were thanked for participating before they were routed back to Prolific to complete their session.

## Results

### Hits and false alarms

The datasets are available at https://osf.io/tymd2/. We examined the effects of within-person variability and expectations of encounter on hits and false alarms made by participants on the bar-admittance task block. In the preregistration (https://osf.io/fsehj/), the hypotheses were based on overall sightings (i.e., a combination of hits and false alarms). This analysis was not included because this variable provided less clarity and no additional information than the separate analyses of hits and false alarms. The analyses on overall sightings can be found on the project’s homepage at Open Science Framework (https://osf.io/tymd2/). Additionally, we reported overall sighting rates within each experimental condition in Table 2. Hits were subjected to a 2 (within-person variability: low or high) × 2 (encounter expectations: low—20% or high—90%) between-subjects ANOVA. The effect of within-person variability on hits was not significant, *F*(1,347) = 3.85, *p* = 0.051, *η*^*2*^_*p*_ = 0.01. Participants in the low variability condition (*M* = 2.35, SE = 0.09) made a similar number of hits as participants in the high variability condition (*M* = 2.09, SE = 0.09). In addition, the effect of expectations of encounter was not significant, *F*(1,347) = 0.34, *p* = 0.558, *η*^*2*^_*p*_ < 0.01. Participants made a similar number of hits across low (*M* = 2.18, SE = 0.89) and high (*M* = 2.26, SE = 0.09) expectation conditions. This finding is inconsistent with results from Moore et al. (2018), in which they found that hits were higher among people with accurate context expectations. Finally, the interaction was not significant, *F*(1,347) = 1.36, *p* = 0.244, *η*^*2*^_*p*_ < 0.01.

**Table 2:** "Experiment one": means and standard deviations for bar-admittance task measures

	High variability		Low variability	
Expectations of encounter	High (*n* = 87)	Low (*n* = 91)	High (*n* = 83)	Low (*n* = 90)
Overall Sightings	3.22 (2.45)	2.93 (1.96)	2.83 (1.30)	2.73 (1.83)
Hits	2.06 (1.17)	2.13 (1.27)	2.46 (1.11)	2.23 (1.23)
False Alarms	1.16 (2.10)	0.80 (1.43)	0.37 (0.88)	0.50 (1.33)
Response Bias (*c*)	0.91 (0.41)	0.94 (0.43)	0.90 (0.34)	0.96 (0.41)
Sensitivity (*d′*)	1.91 (0.83)	2.03 (0.85)	2.35 (0.76)	2.20 (0.83)

Values in parentheses represent standard deviations. Response bias and sensitivity were calculated based on the log linear-transformed hit and false alarm rates

Next, false alarms were subjected to a 2 (within-person variability: low or high) × 2 (encounter expectations: low—20% or high—90%) between-subjects ANOVA. Participants in the high variability condition (*M* = 0.98, SE = 0.11) made more false alarms than participants in the low variability condition (*M* = 0.44, SE = 0.11), *F*(1,347) = 11.51, *p* < 0.001, *η*^*2*^_*p*_ = 0.03. The effect of expectations of encounter on false alarms was not significant, *F*(1,347) = 0.52, *p* = 0.470, *η*^*2*^_*p*_ < 0.01. Contrary to our prediction, false alarms made by participants in the low expectation condition (*M* = 0.65, SE = 0.11) did not differ significantly from false alarms made by participants in the high expectation condition (*M* = 0.77, SE = 0.12). Finally, the interaction was not significant, *F*(1,347) = 2.28, *p* = 0.132, *η*^*2*^_*p*_ < 0.01.

For each of the targets except Target 4, the similarity ratings between the study and test images were higher in the low variability condition than the high variability condition. Similarity between study and test images impacts face perception and memory (Honig et al., 2022; Kramer et al., 2020; Sandford & Ritchie, 2021). Target 4’s study–test images did not vary in similarity by variability condition. Therefore, to conduct a purer test of the impact of variability in Experiment 1, we conducted a logistic regression to determine whether variability impacted sightings of Target 4 alone. The results revealed that within-person variability is a significant predictor of sighting rates, *χ*^2^_(1)_ = 9.94, *p* = 0.002. The difference in odds between the low variability condition and the high variability condition was 1.97, *b* = *0.68, 95% *CI [0.25, 1.10]. In other words, participants in the low variability condition were less likely to make a sighting (predicted probability: 23.74%) than participants in the high variability condition (predicted probability: 27.59%). This finding suggests that exposure to high variability may increase hits (as compared to low variability) when variability is manipulated while holding similarity constant. However, we cannot assess whether exposure to variability impacted false alarms or signal detection measures while holding similarity constant.

### Signal detection measures

We transformed hit and false alarm rates using the log linear method described by Hautus (1995; see also Stanislaw & Todorov, 1999) to eliminate extreme hit and false alarm rates (e.g., *p* = 0 or 1). Specifically, we added 0.5 to all hits and false alarms and divided them by the total number of target trials *plus 1* for calculations of hit rates and by the total number of nontarget trials *plus 1* for calculations of false alarm rates. Then, we calculated discriminability (*dʹ*) as the standardized difference between the *transformed* hit and false alarm rates:

$$d{\prime}=Z(\text{hit rate}) - Z(\text{false alarm rate})$$

Similarly, we calculated response criterion (*c*) based on the *transformed* hit and false alarm rates as:

$$c =\frac{Z(\text{hit rate}) + Z(\text{false alarm rate})}{-2}$$

Discriminability (*dʹ*) and response criterion (*c*) scores are displayed in Table 2. A between-subjects ANOVA was conducted to examine *dʹ.* There was a significant effect of within-person variability, *F*(1,347) = 12.42, *p* < 0.001, *η*^*2*^_*p*_ = 0.03. Contrary to our predictions and findings from the previous research (Juncu et al., 2020), participants in the low variability condition (*M* = 2.28, SE = 0.06) were better at discriminating between the targets and the non-targets than those in the high variability condition (*M* = 1.97, SE = 0.06). The effect of expectations of encounter was not significant, *F*(1,347) = 0.02, *p* = 0.876, *η*^*2*^_*p*_ < 0.01. Discriminability did not differ by expectations of encounter (high expectation: *M* = 2.13, SE = 0.06; low expectation: *M* = 2.12, SE = 0.06). The interaction between within-person variability and expectations of encounter was not significant, *F*(1,347) = 2.48, *p* = 0.116, *η*^*2*^_*p*_ < 0.01.

Next, response criteria (*c*) were subjected to a between-subjects ANOVA. Results showed that response criteria did not differ between participants in the high variability condition (*M* = 0.93, SE = 0.03) and participants in the low variability condition (*M* = 0.93, SE = 0.03), *F*(1,347) < 0.01, *p* = 0.970, *η*^*2*^_*p*_ < 0.01. Similarly, the effect of expectations of encounter was not significant, *F*(1,347) = 0.90, *p* = 0.344, *η*^*2*^_*p*_ < 0.01. There was no evidence that participants in the high expectation condition (*M* = 0.91, SE = 0.03) adopted a more liberal response criterion than those in the low expectation condition (*M* = 0.95, SE = 0.03). Finally, within-person variability did not interact with expectations of encounter, *F*(1,347) = 0.16, *p* = 0.689, *η*^*2*^_*p*_ < 0.01.

### Prospective memory cost

A paired-samples* t*-test was conducted to compare participants’ performance between the ongoing only task block and the bar-admittance task block. Results showed that participants’ ongoing task accuracy was lower on the bar-admittance task block (*M* = 0.72, SD = 0.10), compared to the ongoing only task block (*M* = 0.75, SD = 0.09), *t*(350) = 5.65,* p* < 0.001, Cohen’s *d* = 0.30. Likewise, response times on the bar-admittance task block (*M* = 1394.05, SD = 697.70) were longer than response times on the ongoing only task block (*M* = 1202.81, SD = 432.01), *t*(350) = 5.91, *p* < 0.001, Cohen’s *d* = 0.32. Given that response time data was positively skewed and thus violated the assumption of normality, we used a logarithmic transformation and conducted a *t*-test on the log-transformed response times to verify these results. This analysis confirmed our previous finding that participants responded more slowly on average on the bar-admittance task block, where they had to simultaneously engage in the ongoing task and the bar-admittance task, *t*(350) = 9.90, *p* < 0.001, Cohen’s *d* = 0.28. These findings suggest that participants were dividing their attention between the ongoing task and the prospective memory task as they slowed down on the ongoing task when it was accompanied by the prospective memory task.

As in prospective memory research, we examined attention allocation to the prospective memory task by examining participants' response times on the ongoing task. To test our preregistered hypothesis that high expectations of encounter would result in more costs (i.e., longer response time), we analyzed raw response times on the bar-admittance task block. A 2 (within-person variability: low or high) × 2 (encounter expectations: low—20% or high—90%) between-subjects ANOVA revealed no significant findings. Response times on the bar-admittance task block did not differ by expectations of encounter (high expectation: *M* = 1447.99, SE = 53.49; low expectation: *M* = 1341. 95, SE = 51.83), *F*(1,347) = 2.03, *p* = 0.155, *η*^*2*^_*p*_ < 0.01, or within-person variability (high variability: *M* = 1433.22, SE = 52.27; low variability: *M* = 1356.72, SE = 53.05), *F*(1,347) = 1.05, *p* = 0.305, *η*^*2*^_*p*_ < 0.01. Further, the interaction was not significant, *F*(1,347) = 0.37,* p* = 0.542, *η*^*2*^_*p*_ < 0.01. Considering the violation of normality assumption related to raw response times, we conducted the same analysis with the logarithmic transformed response times as the dependent variable. However, the results again revealed no significant findings, *F*s(1,347) < 3.40, *p*s > 0.066, *η*^*2*^_*p*_* s* < 0.01. Overall, these findings suggest that participants’ attention allocation was not affected by their expectations of encountering the targets or within-person variability.

### Face recognition performance

Participants completed a face recognition task after the bar-admittance task to assess their retrospective memory of the targets’ faces. We conducted a between-subjects ANOVA on participants’ performance on the face recognition task. There was a significant effect of within-person variability on people’s retrospective memory of the targets, *F*(1,347) = 5.49, *p* = 0.020, *η*^*2*^_*p*_ = 0.02. Consistent with our findings on discriminability, participants in the low variability condition (*M* = 2.23, SE = 0.15) recognized more targets on the face recognition test than participants in the high variability condition (*M* = 1.73, SE = 0.15). On the other hand, expectations of encounter did not affect retrospective memory of the targets’ faces (high expectation: *M* = 2.03, SE = 0.15; low expectation: *M* = 1.92, SE = 0.15), *F*(1,347) = 0.26, *p* = 0.608, *η*^*2*^_*p*_ < 0.01. The interaction was not significant, *F*(1,347) = 1.45, *p* = 0.229, *η*^*2*^_*p*_ < 0.01.

We also examined participants’ false alarms on the face recognition task. People in the high variability condition (*M* = 0.38, SE = 0.04) made more false alarms than people in the low variability group (*M* = 0.16, SE = 0.45), *F*(1,347) = 11.42, *p* < 0.001, *η*^*2*^_*p*_ = 0.03. The effect of expectations of encounter was not significant, *F*(1,347) = 0.57, *p* = 0.453, *η*^*2*^_*p*_ < 0.01. The high expectation condition (*M* = 0.24, SE = 0.05) made a similar number of false alarms as the low expectation condition (*M* = 0.29, SE = 0.04). The interaction was not significant, *F*(1,347) = 0.02, *p* = 0.893, *η*^*2*^_*p*_ < 0.01. The face recognition task consisted of the same photographs participants saw during the bar-admittance task, so it is possible that performance was affected by participants' memory of the photographs.

### Post-Task survey

Descriptive statistics of participants’ responses on the post-task survey are shown in Table 3. We conducted exploratory analyses on a subset of these variables to gauge participants’ estimations of their performance on the bar-admittance task. The ANOVA results showed that participants in the high expectation condition (*M* = 3.75, SE = 0.05) reported a higher likelihood (1 = not likely at all, 4 = extremely likely) of believing that they would encounter the targets (i.e., likelihood of encounter) than participants in the low expectation condition (*M* = 2.76, SE = 0.05), *F*(1,347) = 195.87, *p* < 0.001, *η*^*2*^_*p*_ = 0.36. Additionally, participants in the high expectation condition (*M* = 2.46, SE = 0.08) believed that more targets were displayed during the bar-admittance task (i.e., banned persons appearances) than participants in the low expectation condition (*M* = 2.23, SE = 0.08), *F*(1,347) = 4.24, *p* = 0.040, *η*^*2*^_*p*_ = 0.01. Finally, participants in the high expectation condition (*M* = 2.35, SE = 0.08) estimated that they noticed more targets during the bar-admittance task (i.e., banned persons noticed) than participants in the low expectation condition (*M* = 2.12, SE = 0.08), *F*(1,347) = 4.41, *p* = 0.037, *η*^*2*^_*p*_ = 0.01.

**Table 3:** Mean and standard deviation post-task responses in "Experiment one"

Expectations of encounter	High variability		Low variability		Marginal mean: expectations		Marginal mean: variability	
	High	Low	High	Low	High	Low	High	Low
Likelihood of encounter	3.72 (0.58)	2.79 (0.77)	3.77 (0.48)	2.73 (0.75)	3.75 (0.53)	2.76 (0.76)	3.25 (0.83)	3.23 (0.82)
Motivation to search	3.92 (0.38)	3.89 (0.31)	3.34 (0.24)	3.88 (0.33)	3.93 (0.32)	3.88 (0.32)	3.90 (0.35)	3.91 (0.29)
Banned persons appeared	2.46 (1.08)	2.23 (1.02)	2.46 (1.02)	2.23 (1.01)	2.46 (1.04)	2.23 (1.01)	2.34 (1.05)	2.34 (1.01)
Banned persons noticed	2.25 (1.05)	2.10 (1.03)	2.45 (1.00)	2.13 (1.07)	2.35 (1.05)	2.12 (1.03)	2.17 (1.04)	2.28 (1.05)

### Discussion

We expected that high expectations of encounter and high within-person variability would enhance prospective person memory. Surprisingly, low within-person variability led to fewer false alarms and higher discriminability than high within-person variability (cf. Juncu et al., 2020; Sweeney & Lampinen, 2012; Experiment 1). This may be due to the images in the low variability condition being rated as more similar to the test image than the images in the high variability condition. Said another way, this finding may be due to people having a better idea of the targets’ appearance from low variability photographs than from high variability photographs. Indeed, we found the expected effect of variability on sightings for the target whose study–test similarity ratings did not vary by variability condition. As to our null findings regarding expectations of encounter, it is possible that participants had high-baseline expectations of encounter. The context of laboratory-based studies naturally suggests that there is a high probability of target encounter. This may have mitigated the effect of inducing expectations of encounter on participants’ performance. Indeed, participants in the low expectation condition on average reported their belief in the likelihood of encountering the targets to be closest to “somewhat likely,” the third highest point on the 4-point Likert scale. In real-world settings, the spatial and temporal location of a target person is less predictable, which is accompanied by lower expectations of encounter (Moore et al., 2016). Therefore, we tested the effects of expectations of encounter and within-person variability in a field-based study in Experiment two.

## Experiment two

In Experiment 1, we tested the effect of imposed expectations of encounter and exposure to within-person variability at study on prospective person memory in a laboratory-based prospective person memory paradigm. In laboratory-based prospective person memory paradigms, people tend to perform relatively well (Juncu et al., 2020; Lampinen & Sweeney, 2014; Moore et al., 2018; O’Brien & Thorley, 2021) though far from perfect. In contrast, in field-based paradigms people tend to perform remarkably poorly with between 5 and 10% of participants making a sighting despite everyone having an opportunity to sight the target person following exposure to the alert (Lampinen & Moore, 2016a; Lampinen et al., 2016; Moore & Lampinen, 2019; Moore et al., 2016). One reason for this poor performance is that people do not expect to encounter the target person, which leads to them failing to notice the target person in their midst (Moore et al., 2016). Further, the task of being on the lookout for an unfamiliar person is much more complicated and difficult than we can simulate in the laboratory. Therefore, it is important to test the impact of imposed expectations and within-person variability in the field-based setting. In particular, we were concerned that people’s low-baseline expectations and the difficulty of the field-based task may mitigate any benefit that within-person variability could confer upon prospective person memory.

We hypothesized that high variability photographs would result in more total accurate sightings (reported and non-reported) than low variability photographs. We did not expect variability to affect total inaccurate sightings. We hypothesized that high expectations of encounter would lead to more total accurate and inaccurate sightings than low expectations of encounter. Finally, we hypothesized within-person variability and expectations of encounter to interact. As in Experiment one, we maintained competing hypotheses in which variability would only have an effect on sightings when expectations were either high or low. Our hypotheses for Experiment two were developed and preregistered in advance of obtaining the findings from Experiment one, and therefore, they were not influenced by the unexpected findings from Experiment one.

### Method

#### Design

We report how we determined our sample size, all data exclusions (if any), all manipulations, and all measures in the study. A 2 (within-person variability: low or high) × 2 (encounter expectations: low or high) between-subjects design was used. Participants were recruited to a study that they believed was designed to assess their perception of TikTok videos. Participants watched two TikTok videos, one of which was a mock missing persons alert. The dependent variables were total accurate sightings (i.e., accurate reported and non-reported sightings), reported sightings (i.e., sightings reported during the search contest on the sighting survey), non-reported sightings (i.e., sightings recorded in the follow-up survey), lineup identification accuracy (i.e., proportion of accurate lineup identifications of targets in the follow-up survey), and inaccurate lineup identifications (i.e., proportion of inaccurate lineup identifications of targets). The study was preregistered to OSF prior to data collection (https://osf.io/c6fe5).

#### Participants

A power analysis indicated that 394 participants were needed to detect a small effect (e.g., *odds ratio* = 1.77) at *α* = 0.05 and 1 − *β* = 0.80 for a binary logistic regression. Four hundred and four participants were recruited from a southern public university’s participant pool. Based on our preregistered exclusion criteria, 36 participants were excluded from analysis for the following reasons: not watching the TikTok videos or experiencing technical difficulties during the survey (*n* = 12), failing to walk past the target in the hallway while leaving the building (*n* = 8), having exposure to the target before the study (*n* = 6), taking screenshots of the TikToks or attempting to search for the TikToks online (*n* = 4), hurrying through the study (*n* = 3), asking a participant where the target was located (*n* = 1), not speaking fluent English (*n* = 1), and failing to provide an accurate summary of the TikTok videos (*n* = 1). Thus, 368 participants were included in the final sample. We discontinued data collection before we reached our sample size goal because to collect the remaining participants, we would have needed to recruit and intensively train a new research team. This would have resulted in a severely imbalanced sample size per target. We used an a priori power analysis to reverse engineer our power estimates and determined that with 368 participants, we were powered at 0.773 to detect a small effect. Participants received course credit for participating and had the chance to win a portion of a $300 prize for an accurate sighting and one of five $50 gift cards for their participation. The mean age of participants was 19.57 (SD = 1.43, Range: 18–29). Two hundred and fifty-six participants (69.57%) identified as female, 106 (28.80%) identified as male, five (1.36%) identified as gender variant/nonconforming, and one (0.27%) preferred not to respond. By ethnicity, 251 (68.21%) participants identified as White, 38 (10.33%) participants identified as two or more ethnicities, 28 (7.61%) participants identified as Hispanic/Latinx, 21 (5.71%) participants identified as Black/African-American, 17 (4.62%) participants identified as Native American or Alaska Native, 11 (2.99%) participants identified as Asian, one (0.27%) participant identified as Native Hawaiian or other Pacific Islander, and one (0.27%) participant preferred not to respond.

#### Materials

*Targets and similarity ratings* Three female research assistants acted as targets (i.e., the mock missing person) for the study. Of these three targets, target one was white and 20 years old (*n* = 289), target two was white and 20 years old (*n* = 41), and target three was Latinx/Hispanic and 19 years old (*n* = 38) (see Appendix C). The targets were selected based upon their willingness to act in this role and their availability as we had to coordinate their schedules to match the schedules of the research assistants who ran the sessions. Each target provided between eight and ten ambient photographs of themselves that met the criteria for low and high variability photographs used in Experiment one. We took portrait, mugshot-style photographs of each target that showed the full, front view of their face with a neutral expression.

We assessed the similarity of the target photographs during the second pilot study described in Experiment one. Photograph pairs that had an average similarity rating above three were used as low variability study photographs, and photograph pairs that had an average rating below three were used as high variability study photographs. Table 4 shows the average similarity ratings between the final Experiment two target photographs.

**Table 4:** Mean similarity ratings of the target photographs in Experiment two

	Low variability			High variability		
Target number	1	2	3	1	2	3
*Study images*						
Study image one and two	4.50	3.47	4.23	3.27	3.07	2.36
Study image one and three	4.70	3.93	4.53	3.80	2.53	3.43
Study image two and three	4.27	4.60	3.90	3.00	2.63	2.90
Grand mean	4.49	4.00	4.22	3.36	2.74	2.90
*Study and test images*^a^						
Study image one and test	2.83	2.67	3.57	2.77	3.47	2.57
Study image two and test	2.87	2.83	3.57	2.97	2.93	2.80
Study image three and test	2.60	2.50	3.13	2.67	2.40	2.50
Grand mean	2.77	2.67	3.42	2.80	2.93	2.62

^a^The test image of the target for this study was a representative image of the target taken at the beginning of the study. In reality, the appearance of the target at test may have varied because of the nature of the study

**Fig. 2:**
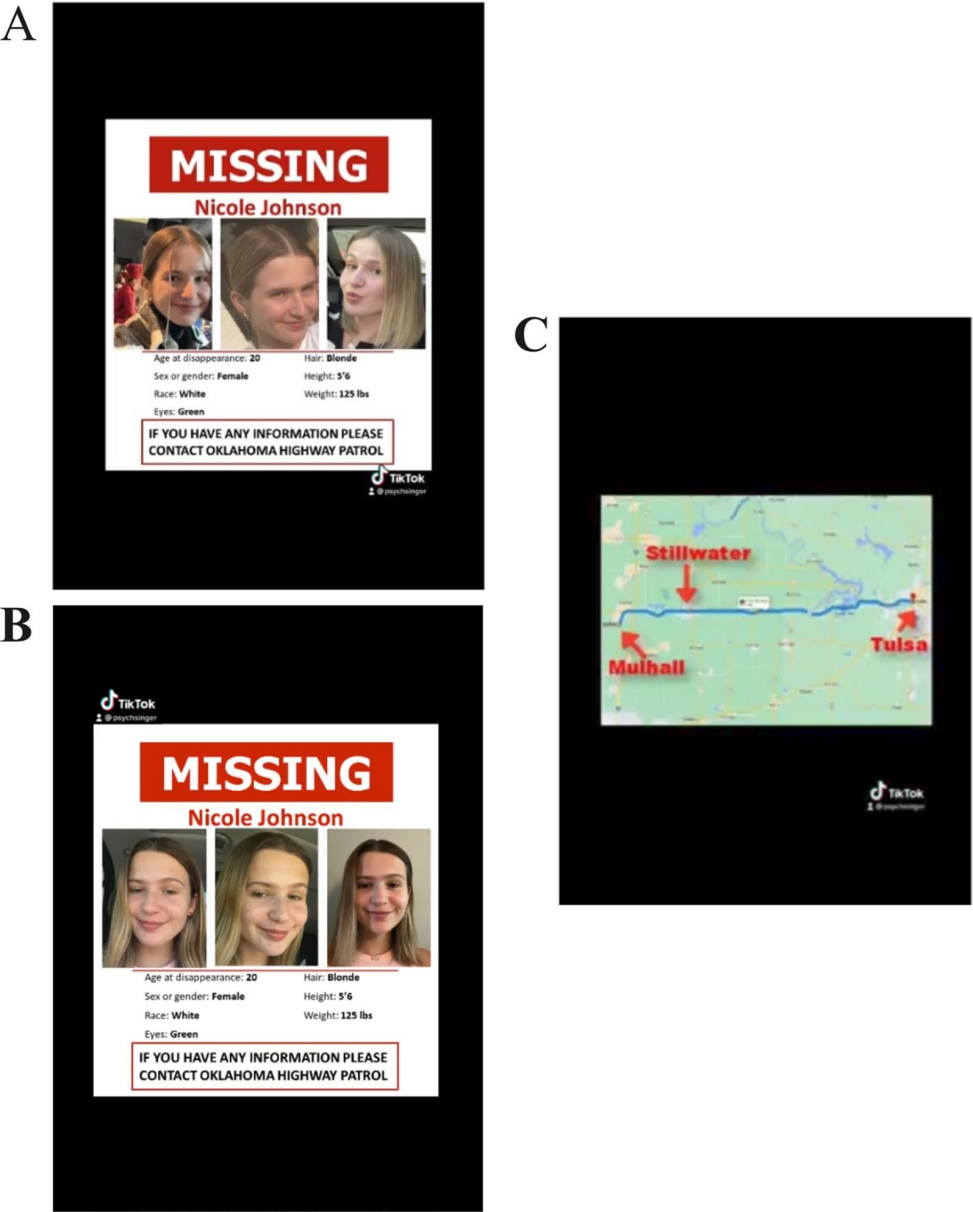
High and low variability posters and map screenshot from the missing persons alert TikTok for target one. *Note.* Image **A** (high variability) or **B** (low variability) is shown for 21 s before image **C** appears for 15 s. The TikTok then switches back to image **A** or **B**, respectively, for the remaining 13 s.

*TikTok missing person alert* We created a TikTok-style video to serve as the missing persons alert. Two versions of the TikTok video were created for each target: one version containing the low variability photographs of the target and one version containing the high variability photographs of the target. The TikTok began with a female narrator asking viewers to stop and pay attention as she had an important message to share. A missing person poster with the target's low or high variability photographs and demographic information was shown as the background. The narrator stated that a young woman named *Nicole Johnson* was reported missing from a nearby town after she failed to appear for a doctor’s appointment. The narrator said it was likely that *Nicole* passed through the town where we conducted the study and that her family was worried something may have happened to her. A map with *Nicole’s* route was shown as the background image with labels indicating each location along the route to emphasize that *Nicole* may have gone missing nearby. The narrator asked viewers to study the missing persons poster and to report sightings to the State Highway Patrol. The TikTok is approximately 50 s in duration with the missing person’s images displayed for a total of 34 s. Figure 2 shows screenshots of the TikTok shown to participants.

*TikTok survey* We created a Qualtrics survey to expose participants to and gauge participants’ reactions to two TikTok videos, a TikTok reviewing a local restaurant and the mock missing person alert TikTok. The survey included a statement that the TikToks had been posted within the past year. First, the restaurant TikTok was displayed with a timer that prevented advancing to the next page until the video was over. Then, there was an attention check question that requested a summary of the main points of the TikTok. The survey contained questions about how important the information conveyed in the TikTok was, how concerning the information was, how clear the presenter’s tone was, and how well the presenter did on a 6-point Likert scale (1 = extremely poorly, 6 = extremely well). Additionally, there were questions that assessed whether the TikTok was viewed and whether any technical difficulties occurred with viewing the TikTok. We used an emotional experience questionnaire to assess the following emotions: anxious, happy, frightened, angry, surprised, interested, disgusted, sad, and hopeful on a 4-point Likert scale (1 = not at all, 4 = extremely). There were five questions about the quality of the TikToker and TikTok story on a 5-point Likert scale (1 = strongly disagree; 5 = strongly agree).

The same process occurred for the missing person alert TikTok with the following exceptions and additions. The survey contained all 6 missing person TikToks (high and low variability versions for each of the 3 targets), and we set up the survey structure to show one TikTok to each participant by randomly assigning them to see one version of their target’s TikTok (high or low variability) based on which target was scheduled for the session. Following the missing person TikTok and questions described above, a statement was shown stating that the person featured in the TikTok was not actually missing and that the TikTok was created for the study. The statement indicated that law enforcement should not be contacted in the case of sightings of the target and that, instead, we were hosting a contest to find the person. If participants spotted the person featured from the TikTok within the next week on campus they could report their sighting to an easy to remember email address set up for the study for a chance to win a portion of a $300 cash prize. Participants were informed that they would be expected to email to report a sighting within 24 h of the sighting to indicate where and when they saw the target person and what that target person was wearing.

The survey was coded to randomly assign one of the expectations of encounter conditions. Specifically, the survey included text that stated that based on prior research, there was a 20% (i.e., low expectations of encounter) or 90% (i.e., high expectations of encounter) chance that the target would be on campus in the next week. The survey contained comprehension check questions to ensure that participants knew that the person from the missing person TikTok was not actually missing, who should be contacted if they spot the target, what email address sightings be reported to, the amount of prize money, and the percent chance of the target appearing on campus. The survey also contained a demographic questionnaire. Finally, participants answered self-reported questions about their expectations of encountering the target person sometime in the next week on a 5-point scale (1 = unlikely, 5 = very likely) and the degree to which they have formed specific intent to search for the target person on a 4-point scale (1 = not at all, 4 = very much so). Participants were informed that they would receive a follow-up survey 24 h after the laboratory session. Then the survey concluded.

*Follow-up survey* A follow-up survey was created in Qualtrics for administration 24 h after the study session to assess participants' memory of the missing person TikTok and whether participants made a sighting of the target but did not report it (i.e., non-reported sightings). The survey included questions about the extent to which participants looked for the target (1 = not at all, 4 = very much so), whether they believed that they had a chance to encounter the target, how likely they believed it was that they would encounter the target (1 = not at all likely, 4 = extremely likely), whether they remembered to look for the target, and what (if anything) would have helped to remind the participant to look for the target.

*Non-reported sightings* The follow-up survey also included a question about whether participants thought they had seen the target but did not report it to researchers. If so, follow-up questions were asked about the target’s appearance, clothing, location, and the date and time they were encountered. These questions allowed us to assess the accuracy of the sighting. The final questions about the non-reported sighting were why the participant did not report the sighting and how confident the participant was that the person they spotted was the target.

Additionally, the survey contained open-ended questions that asked about the target’s name, the reason the police were looking for the target, and the target’s appearance. The survey contained a six person, simultaneous lineup that included a new, mugshot-style photograph of the target and five look-alike fillers to assess retrospective person memory. Finally, there were questions about whether participants found the target on their own and whether they shared the target’s location with other participants. The debriefing explained the purpose of the study and thanked participants for their participation. The research team checked with targets each week to verify whether the non-reported sightings were accurate.

*Sighting survey* A sighting survey was created in Qualtrics for participants to report sightings of the target (i.e., reported sightings). The sighting survey contained the same questions as the non-reported sightings questions on the follow-up survey.

#### Procedure

Participants were informed that they were participating in a study designed to assess the quality and content of TikTok videos. Participants provided informed consent and began the TikTok Survey on their own in sessions of one to four participants. Meanwhile, the research assistant running the session notified the target, who was stationed in a separate room, to move to the designated sighting location, which was approximately 30 feet from the laboratory in a hallway, to ensure that participants had the opportunity to encounter the target as they left the laboratory.

After completing the TikTok survey, the research assistant handed each participant a paper debriefing and the contest rules. This provided an excuse to release participants one at a time so each participant had a chance to see the target in the hallway, while avoiding instances where another participant might give away the target’s location. Targets were trained to stand with their back against the wall in the designated sighting location and to look at their phone without acknowledging the participant as they walked past. Targets returned to their laboratory room several minutes after the last participant passed them in the hallway to ensure that they did not rouse suspicion among participants and that participants for the next session did not see them. While we orchestrated a designated sighting opportunity for participants, the targets were undergraduate students at the institution we collected data from, and, thus, participants may have had other opportunities to make a sighting of the target. Approximately 24 h after the TikTok survey was completed, we sent the follow-up survey to participants. We sent reminders to complete the Follow-Up Survey eight, 24, and 48 h after the follow-up survey was sent to participants. Participants were sent the sighting survey automatically if they reported a sighting to the sighting email address.

## Results

### Reactions to TikTok videos

The datasets are available at https://osf.io/tymd2/. We compared participants’ reactions to the missing person TikTok and the Restaurant TikTok using paired-samples *t*-tests. Due to running multiple comparisons, we applied a Bonferroni correction to control for familywise error rates (adjusted α: 0.05/18 = 0.003). Test statistics and effect sizes are shown in Table 5. There was a significant difference in participants’ rated importance of the content, how concerned, anxious, happy, frightened, angry, surprised, disgusted, and sad they felt about the content, how interesting they rated the content to be, and the pace and confidence ratings of the narrator of the TikTok stories, all *p*s < 0.002.

**Table 5:** Experiment two: reactions to TikTok videos

	Missing person TikTok		Restaurant TikTok		*t*	Cohen’s *d*
	*M*	SD	*M*	SD		
Importance	5.84	0.66	3.13	1.12	40.64*	2.12
Concerned	5.26	0.87	1.77	1.20	50.26*	2.62
Clarity	5.51	0.63	5.58	0.64	− 1.77	− 0.09
Anxious	2.69	0.84	1.05	0.30	36.50*	1.91
Happy	1.00	0.00	2.42	0.85	− 31.87*	− 1.66
Frightened	2.50	0.90	1.00	0.05	32.04*	1.67
Angry	1.52	0.73	1.01	0.07	13.49*	0.70
Surprised	2.02	0.98	1.74	0.92	4.55*	0.24
Interested	3.03	0.83	2.98	0.85	0.79	0.04
Disgusted	1.51	0.82	1.03	0.21	11.35*	0.59
Sad	2.89	0.95	1.01	0.09	38.23*	1.99
Hopeful	1.82	0.86	1.64	0.80	3.31*	0.17
Good pace	4.23	0.90	3.95	0.97	5.13*	0.27
Interesting	4.34	0.90	3.42	1.04	15.95*	0.83
Good job	5.18	0.80	5.13	0.78	0.93	0.05
Appropriate	4.33	0.91	4.39	0.83	− 1.15	− 0.06
Clear language	4.56	0.69	4.51	0.75	1.27	0.07
Confident	4.03	0.96	4.49	0.77	− 9.11*	− 0.47

*Significant after the Bonferroni correction

### Reported sightings

Twenty-four participants (6.52%) emailed to report sightings of the target (i.e., reported sightings) and completed the sighting survey. Fourteen (58.33%) of these sightings were correct and 10 (41.67%) were incorrect. The number of reported sightings made by participants within each experimental condition appears in Tables 6 and 7.

**Table 6:** Experiment two: sighting frequencies and percentages by experimental conditions among participants who made a sighting

Expectations of encounter	High variability		Low variability	
	High	Low	High	Low
Accurate sightings	9 (40.91%)	4 (22.22%)	10 (38.46%)	4 (20.00%)
Reported sightings	5 (22.73%)	2 (11.11%)	5 (19.23%)	2 (10.00%)
Non-reported sightings	4 (18.18%)	2 (11.11%)	5 (19.23%)	2 (10.00%)
Inaccurate sightings	13 (59.09%)	14 (77.78%)	16 (61.54%)	16 (80.00%)
Reported sightings	4 (18.18%)	0 (0.00%)	3 (11.54%)	3 (15.00%)
Non-reported sightings	9 (40.91%)	14 (77.78%)	13 (50%)	13 (65.00%)
Total sightings	22 (100%)	18 (100%)	26 (100%)	20 (100%)

**Table 7:** Experiment two: sighting frequencies and percentages by experimental conditions among all participants

	High variability		Low variability	
Expectations of encounter	High	Low	High	Low
Accurate sightings	9 (10.23%)	4 (4.21%)	10 (10.99%)	4 (4.26%)
Reported sightings	5 (5.68%)	2 (2.11%)	5 (5.49%)	2 (2.13%)
Non-reported sightings	4 (4.55%)	2 (2.11%)	5 (5.49%)	2 (2.13%)
Inaccurate sightings	13 (14.77%)	14 (14.74%)	16 (17.58%)	16 (17.02%)
Reported sightings	4 (4.55%)	0 (0.00%)	3 (3.30%)	3 (3.19%)
Non-reported sightings	9 (10.23%)	14 (14.74%)	13 (14.29%)	13 (13.83%)
Total sightings	22 (25%)	18 (18.95%)	26 (28.57%)	20 (21.28%)

### Non-reported sightings

Three hundred and nineteen participants (86.68%) completed the follow-up survey. On average, the time difference between the completion of the initial TikTok survey and the follow-up survey was 47.76 h (SD = 54.15). Among the participants who completed the follow-up survey, 19 participants made reported sightings and therefore, were less likely to make additional non-reported sightings. However, three out of the 19 participants who made reported sightings also made additional non-reported sightings. Their non-reported sightings were redundant with their reported sightings and therefore were not counted toward non-reported sightings. For the rest of the participants who completed the follow-up survey (*n* = 300), there were 62 (20.67%) non-reported sightings. Importantly, 13 (20.97%) of these non-reported sightings were correct and 49 (79.03%) were incorrect sightings (see Table 6 for the number of non-reported sightings within each cell).

### Total accurate sightings

As put forth in our preregistration and following the work of Moore and Lampinen (2019), we examined the effect of within-person variability and expectations of encounter on total accurate sightings. Our preregistration indicated that we would dummy-code our categorical variables. However, we decided to use effect coding instead for ease of interpretation of the models that included interaction terms. We first entered expectations of encounter (− 0.5 = low expectations; 0.5 = high expectations) as the predictor in a logistic regression model. Results showed that this model was significantly better than the null, intercept-only model, *χ*^*2*^_(1)_ = 5.64, *p* = 0.018. As hypothesized, participants in the high expectation condition (predicted probability: 10.61%) were 2.69 times more likely to make an accurate sighting compared to the low expectation condition (predicted probability: 4.23%), *b* = 0.99, *95% *CI [0.14, 1.84]. Next, we entered within-person variability (− 0.5 = low variability; 0.5 = high variability) as the predictor, along with expectations of encounter. Contrary to our hypothesis, this model was not significantly better than the previous model, *χ*^*2*^_(1)_ = 0.02, *p* = 0.884. Holding expectations of encounter constant, participants were equally likely to make an accurate sighting across the within-person variability conditions (low: 6.94%; high: 6.57%), *b* = − 0.06, *95% *CI [− 0.85, 0.73]. Finally, a full logistic regression model was constructed, including expectations of encounter, within-person variability, and their interaction as the predictors. Results showed that the full model was not significantly better than the first model, *χ*^*2*^_(2)_ = 0.03, *p* = 0.986. Expectations of encounter did not interact with within-person variability.

### Total inaccurate sightings

We also examined the effects of expectations of encounter and within-person variability on the probability of making an inaccurate sighting. We entered expectations of encounter as the predictor in the first logistic regression model. The effect of expectations of encounter on total inaccurate sightings was not significant, *χ*^*2*^_(1)_ = 0.01, *p* = 0.932. The predicted probability for the low expectation condition to make an inaccurate sighting (15.87%) was not different from the predicted probability for the high expectation condition (16.62%), *b* = 0.02, *95% *CI [− 0.53, 0.58]. Next, we entered within-person variability as the predictor of total inaccurate sightings. Within-person variability was not a significant predictor of total inaccurate sightings, *χ*^*2*^_(1)_ = 0.44, *p* = 0.506. The predicted probability of participants making an inaccurate sighting did not differ by within-person variability (low: 17.30%; high: 14.75%), *b* = − 0.19, *95% CI*: [− 0.75, 0.37]. Finally, we constructed the full model with both predictors and their interaction as predictors of total inaccurate sightings. The full model was not significantly different from the intercept-only model, *χ*^*2*^_(3)_ = 0.45, *p* = 0.929.

### Face recognition performance

In the follow-up survey, we assessed participants’ retrospective memory of the target’s face by asking them to identify the target from a lineup. Two hundred and fifty-six participants made a selection from the lineup (average retention interval: 46.29 h, SD = 46.16). A logistic regression was conducted to examine the effect of expectations of encounter on lineup identification accuracy. Results showed that this model was not significantly better than the intercept-only model, *χ*^*2*^_(1)_ = 0.06, *p* = 0.808. The predicted probability of a lineup identification being correct did not differ between the low expectation condition (48.03%) and the high expectation condition (46.5%), *b* = − 0.06, *95% *CI [− 0.55, 0.43]. Similarly, results from a separate logistic regression showed no significant effect of within-person variability, *χ*^*2*^_(1)_ = 0.24, *p* = 0.621. The predicted probability of a lineup identification being correct in the low variability condition (45.73%) did not differ from that in the high variability group (48.82%), b = 0.12, *95% *CI [− 0.37, 0.61]. Finally, the full model, which included expectations of encounter, within-person variability, and the interaction term, was not significantly better than the intercept-only model, *χ*^*2*^_(3)_ = 6.01, *p* = 0.111.

### Exploratory analysis

We conducted exploratory analyses to gauge participants’ intent to search and their estimated likelihood to encounter the target from their responses to the TikTok survey *prior to the search*. Search intent (1 = not at all, 4 = very much so) did not differ across experimental conditions, and the interaction was not significant, *F*s(1,364) < 0.97, *p*s > 0.327, η^2^_p_s < 0.01. On the analysis of estimated likelihood to encounter the target (1 = unlikely, 5 = very likely), participants in the high expectation condition (*M* = 3.48, SE = 0.10) estimated their opportunity to spot the target to be higher than participants in the low expectation condition (*M* = 2.87; SE = 0.09), *F*(1, 364) = 19.89, *p* < 0.001, η^2^_p_ = 0.05. Further, there was a significant interaction, *F*(1, 364) = 6.91, *p* = 0.009, η^2^_p_ = 0.02. When within-person variability was low, participants in the high expectation condition (*M* = 3.74, SE = 0.14) estimated their opportunity to spot the target to be higher than participants in the low expectation condition (*M* = 2.78, SE = 0.13), *t*(364) = 5.03, *p* < 0.001. However, when within-person variability was high, participants in the high expectation condition (*M* = 3.22, SE = 0.14) thought they had a similar opportunity to spot the target as participants in the low expectation condition (*M* = 2.97, SE = 0.13), *t*(364) = 1.29, *p* = 0.199.

As in "Experiment one," we also conducted exploratory analyses on participants’ responses *after the search* from the follow-up survey. Participants in the high expectation condition (*M* = 1.85, SE = 0.06) reported that they actively looked for the target more than participants in the low expectation condition (*M* = 1.67, SE = 0.06), *F*(1,314) = 4.66, *p* = 0.032, *η*^2^_*p*_ = 0.01. Additionally, participants in the high expectation condition (*M* = 1.94, SE = 0.06) reported a higher likelihood of believing that they would encounter the target than participants in the low expectation condition (*M* = 1.71, SE = 0.06), *F*(1,314) = 7.68, *η*^2^_*p*_ = 0.02. Within-person variability did not affect participants’ reported looking behavior or the follow-up survey measure of their expectations of encounter. The interactions were also not significant.

## General discussion

Locating missing and wanted persons is important, but accurate sightings in field-based simulated searches are extremely low (Lampinen et al., 2016; Moore et al., 2016; Moore & Lampinen, 2019). Attention and face recognition errors cause a high rate of sighting failures (Lampinen & Moore, 2016a, 2016b; Moore & Lampinen, 2019; Moore et al., 2016). We tested interventions to improve each process, namely expectations of encounter on attention and within-person variability on face recognition, to increase sightings in a laboratory-based and field-based setting. We expected both interventions to increase sightings. We were especially interested in how within-person variability and expectations of encounter interact. The effectiveness of within-person variability may depend on expectations of encounter since these expectations inform search behavior which informs whether a person has a chance to recognize the target. Specifically, we hypothesized that participants with high expectations may benefit from improved face recognition because they are more likely to search for the target than those with low expectations. In contrast, we hypothesized that participants with low expectations may instead benefit from improved face recognition if they were putting some effort into searching. We tested this design in a high-baseline expectation condition (e.g., laboratory-based paradigm, Experiment one) and a low-baseline expectation condition (e.g., field-based paradigm, Experiment two).

In our laboratory-based study Experiment one, we found low within-person variability resulted in fewer false alarms, better discriminability, and better retrospective memory of the target persons’ faces than high within-person variability. This finding may reflect the fact that the study–test images were rated as more similar in the low variability condition than the high variability condition. When we analyzed the target whose study–test images were the same across variability conditions, we found the expected effect of high variability increasing accurate sightings compared to low variability. However, in our field-based study Experiment two, we found no effect of within-person variability on sightings or retrospective memory of the target’s face. These findings are contrary to our hypothesis and previous research suggesting the beneficial effect of high within-person variability on face memory (Juncu et al., 2020; Menon et al., 2015 Experiments 2–3; Ritchie & Burton, 2017, Experiment 1A; Ritchie et al., 2021 Experiments 2–4; Sweeney & Lampinen, 2012; Experiment 1). In Experiment one," in which baseline expectations were anticipated to be high, expectations of encounter did not impact sighting rates, discriminability, or response bias. However, in Experiment two, in which baseline expectations were low (pre-search measure of participants’ expectations of the likelihood that they would have an opportunity to spot the target: *M* = 3.17 (out of 6), *SD* = 1.34), participants with high expectations of encounter were more likely to make an accurate sighting than participants with low expectations of encounter. Finally, in Experiment one neither variable impacted participants' attention allocation to searching for the target persons.

### Expectations of encounter

In Experiment one, we found no evidence that expectations of encounter affected prospective person memory performance. These results contradict findings from the previous laboratory-based research (Moore et al., 2018). However, this discrepancy may be reconciled by considering their methodological differences. Specifically, Moore et al. (2018) manipulated context expectations, in which participants believed they had a higher chance of encountering the targets in one context relative to another context. In our experiments, context was fixed, and participants were told that their chance of encountering the targets was either high or low. Given high-baseline expectations inherent to the laboratory-based prospective memory paradigm, manipulating *relative* expectations across different contexts may be more effective at inducing the intended difference in participants’ expectations in the laboratory than giving them an *absolute* percentage in one context that lasts throughout the study. Despite this difference, our post-study measure of expectations indicated that participants given high expectations believed it was more likely that they would encounter the targets than participants given low expectations. Additionally, there was an effect of expectations of encounter on accurate sightings in Experiment two. This finding replicates previous field-based studies (Lampinen et al., 2016; Moore et al., 2016). Perhaps the reason for the discrepancy in the effect of expectations across paradigms corresponds to participants' relatively high expectations of encounter in the laboratory-based study, the overall ease of the task, or participants adjusting their behavior in response to their experience. Indeed, participants in the laboratory-based study (*M* = 3.24, SD = 0.82) had higher expectations of encounter than participants in the field-based study (*M* = 1.83, SD = 0.74), as measured by self-reported expectations of encounter after the search tasks, *t*(667) = 23.34, *p* < 0.001, Cohen’s *d* = 1.81. Regarding the latter, we theorize that participants given low expectations in the laboratory-based study may have realized that targets were indeed appearing and adjusted their search behavior during the task in accordance with this information (see Scullin et al., 2013 for a related finding in prospective memory).

### Within-person variability

Our research was inspired by work that has found that exposing people to the variability in an unfamiliar person’s appearance can enhance face learning (Juncu et al., 2020; Menon et al., 2015, Experiments 2 and 3; Ritchie & Burton, 2017 Experiment 1A; Ritchie et al., 2021 Experiments 2–4; Sweeney & Lampinen, 2012; Experiment 1). Importantly, exposure to multiple photographs, without controlling for within-person variability, improved accuracy without increasing false alarms in contrast to exposure to a single photograph on a prospective person memory task (Sweeney & Lampinen, 2012; Experiment 1). Additionally, photographs that showcase the variability in a person’s appearance improved discriminability but did not increase response bias on a prospective person memory task (Juncu et al., 2020). However, the research findings on the impact of variability on face perception and face learning are mixed. On simultaneous face matching tasks, some research finds exposure to variability increases match accuracy without decreasing mismatch accuracy (Mileva & Burton, 2019, Experiments 1–2; Menon et al., 2015, Experiment 1; White et al., 2014), while others find that variability decreases mismatch accuracy (Menon et al., 2015, Experiment 3; Ritchie et al., 2021, Experiments 1 and 2). Further, some studies find no benefit of variability on simultaneous face matching (Kramer & Reynolds, 2018; Mileva & Burton, 2019, Experiment 3; Ritchie et al., 2020). The research on face learning is more consistent; finding that exposure to variability improves accuracy (Ritchie & Burton, 2017; Experiment 1A) without a cost (Juncu et al., 2020; Menon et al., 2015, Experiments 2 & 3; Ritchie et al., 2021 Experiments 2–4; Sweeney & Lampinen, 2012, Experiment 1). Only one published study has found no effect of variability on sequential face matching (Ritchie & Burton, 2017 Experiment 1B). We predicted that exposure to high within-person variability would increase discriminability and response bias on a prospective person memory task. However, in Experiment 1 we found that participants in the low variability condition had higher discriminability and made fewer false alarms than participants in the high variability condition. Below we make the case that this finding was due to the similarity ratings between the study and test images being higher in the low variability condition than the high variability condition for three of the four targets in Experiment 1.

### A similarity account

Recent research has found that the similarity between study and test images may have a stronger influence on face perception and memory (Honig et al., 2022; Kramer et al., 2020; Sandford & Ritchie, 2021) than within-person variability. Kramer et al. (2020) examined the influence of within-person variability on simultaneous face matching to targets in CCTV-style footage and found that there was a low variability advantage. Specifically, low variability photographs increased hits and false alarms. The researchers reasoned that this finding was due to the low variability images being taken more recently and thus being more similar to the test footage of the target than the high variability images. Researchers have found that face matching performance is better when images were taken within minutes of each other compared to months apart (Fysh & Bindemann, 2018; Megreya et al., 2013). Sandford and Ritchie (2021) found that the similarity between study and test images influenced face matching more than variability. Using face matching (Experiment 1) and face recognition (Experiment 2) paradigms, Honig et al. (2022) found that high variability study images improved performance in contrast to low variability study images but only when the test images were also high variability. They argue that “learning from high variability is advantageous because perceptually different images of the same identity are more likely to be similar to the learning set” (Honig et al., 2022, p. 8). However, performance was best when the test image resembled the low variability study images. Our findings from Experiment 1 converged with research that finds that similarity has a stronger influence on face memory than variability. In Experiment one, participants in the high variability condition made more false alarms and had lower discriminability than participants in the low variability condition. Like Kramer et al. (2020), we discovered that generally the low variability photographs that participants submitted were taken closer in time to the target test image than the high variability photographs. Additionally, for all but one of the four targets in Experiment one, the low variability study image sets were rated as more similar to the target test image than the high variability study image sets, in contrast this trend did not occur between the study images and lineup image in Experiment two.^3^However, it is worth noting that for Experiment 2 we do not have a pure measure of this as the targets’ appearance at test (sighting) was live and could have varied in similarity between the two variability conditions over time. When considered in this light, our findings from "Experiment one" fit with recent research demonstrating that the similarity between study and test images impacts face perception and memory (Honig et al., 2022; Kramer et al., 2020; Sandford & Ritchie, 2021). Specifically, our findings suggest that when similarity is confounded with variability that similarity overpowers the impact of variability.

When similarity was held constant across variability conditions (i.e., Target 4), we found that participants in the high variability condition were more likely to make a sighting than participants in the low variability condition. To fully understand whether variability and similarity impact prospective person memory, both variables need to be manipulated in field-based research to determine whether their impact in the laboratory generalizes to more realistic, complex conditions. If these interventions are contingent upon similarity between study and test views, this complicates matters for practitioners trying to choose a photograph to feature in a missing or wanted persons alert because the person’s appearance at test is naturally unknown in these situations. However, future research is needed before policy recommendations can be made to practitioners.

## Conclusions

Identifying interventions to improve attention to searching and face recognition is key to improving sighting rates of missing and wanted persons. In the current research, we examined interventions on attention via expectations of encounter and face recognition via within-person variability. Expectations affected performance in realistic person searches, which confirmed that increasing expectations can increase recovery rates of missing and wanted persons. Future research and applied work should explore interventions to combat individuals’ low expectations by, for example, letting searchers know when a missing or wanted person is suspected to be in their vicinity (Moore et al., 2016). As for improving facial recognition, our findings indicated that the impact of variability in the laboratory may be contingent upon its relation to similarity between study and test appearances. Similarity between study and test appearance may explain the beneficial effect of low variability in "Experiment one" and exposure to variability may only be beneficial when similarity is held constant. Our initial research suggests that the impact of variability may not extend to more realistic prospective person memory conditions, but further research on the impact of variability and similarity on prospective person memory is needed to better understand the role of each in performance at searching for a missing or wanted person.

## Low variability photographs

Thank you for consenting and allowing us to use your photographs for research purposes! Your photographs will be used to contribute to scientific research about human face perception and memory.

The first few photographs we would like you to submit are photographs that were taken close in time to one another (i.e., photographs taken of you within the same day or at the same event). In other words, these photographs should show the consistency of your facial appearance. For example, these could be photographs taken of you at one social event or selfies you took of yourself throughout the day.

Here are some example photographs:

Please submit a minimum of four photographs. Additional photographs may be submitted if you wish. Each photograph you submit should adhere to the following criteria:

1. Please submit photographs as a “.jpg” file.
2. Please do not edit or crop your photograph. We will crop photographs to show just your face and will blur the background.
3. Your entire face must be visible (no hats, glasses, sunglasses, hands, or any other objects that obscure your face—unless those items are worn daily for religious purposes, and in such cases those items must not obscure any facial features).
4. Do not submit photographs with extreme or heavy amounts of makeup (e.g., Halloween costumes), visible tattoos on the face and/or upper neck, or ones that contain facial piercings (e.g., nose piercings, eyebrow piercings).
5. Photographs must be clear and in color.
6. The photograph should include your full face (i.e., no masks).
7. Photographs with your face slightly turned to the side are acceptable as long as the majority of your face can be seen.
8. Photographs taken vertically (i.e., up/down, not sideways/horizontally) are preferred but horizontal photographs may be considered.
9. Do not use filters commonly used on social media (e.g., Snapchat).
10. Photographs should not be digitally altered.
11. Ideally the photograph will include just yourself. If other people are present, they should be easily removed or cropped out.

Please indicate the approximate date that each photograph was taken and your approximate age.

## High variability photographs

The next photographs we would like you to submit are photographs that were taken at different times from one another (e.g., photographs taken of you one month ago, 6 months ago, a year ago). In other words, these photographs should show the variability of your appearance over time.

Here are some example photographs:

Please submit a minimum of 4 photographs. Additional photographs may be submitted if you wish. Each photograph you submit should adhere to the following criteria:

1. Please submit photographs as a “.jpg” file.
2. Please do not edit or crop your photograph. We will crop photographs to show just your face and will blur the background.
3. Your entire face must be visible (no hats, glasses, sunglasses, hands, or any other objects that obscure your face—unless those items are worn daily for religious purposes, and in such cases those items must not obscure any facial features).
4. Do not submit photographs with extreme or heavy amounts of makeup (e.g., Halloween costumes), visible tattoos on the face and/or upper neck, or ones that contain facial piercings (e.g., nose piercings, eyebrow piercings).
5. Photographs must be clear and in color.
6. The photograph should include your full face (i.e., no masks).
7. Photographs with your face slightly turned to the side are acceptable as long as the majority of your face can be seen.
8. Photographs taken vertically (i.e., up/down, not sideways/horizontally) are preferred but horizontal photographs may be considered.
9. Do not use filters commonly used on social media (e.g., Snapchat).
10. Photographs should not be digitally altered.
11. Ideally the photograph will include just yourself. If other people are present, they should be easily removed or cropped out.

Please indicate the approximate date that each photograph was taken and your approximate age.

## Standard photograph instructions

The final photographs we would like you to submit are photographs of the style typically used for driver’s license, state IDs, school photographs, mugshots, etc. Please take a new photograph of yourself that meets this criteria. This photograph should show the full, frontal view of your face.

Here is an example photograph:

Please submit a minimum of two photographs. Additional photographs may be submitted if you wish.

Below are recommended steps for taking a neutral photograph similar to those for ID cards, driver’s licenses, etc. Please follow the instructions to take your own neutral photograph, or you may upload any existing photographs that meet this criteria:

1. Please submit photographs as a “.jpg” file.
2. Consider having someone else take the photograph for you or assist you in taking the photograph.
3. Stand or sit straight and remain still while taking the photograph.
4. Photographs must have a plain, uniform background (white or a light colored wall is preferred).
5. Use sufficient lighting that highlights both sides of your face equally and casts no shadows. Avoid extremely bright lighting.
6. Photographs must be free from glare and reflections.
7. Photographs must be clear and in color.
8. With shoulders and face squarely facing forward, face the camera directly, at eye level, centered, before taking the photograph so your full face is visible.
9. Leave space around both sides of your face and above your head in the photograph, so your entire face is captured.
10. Adjust your hair and clothing so that your entire face is visible (no hats, glasses, sunglasses, hands, or any other objects that obscure your face—unless those items are worn daily for religious purposes, and in such cases those items must not obscure any facial features).
11. Do not submit photographs with extreme or heavy amounts of makeup (e.g., Halloween costumes), visible tattoos on the face and/or upper neck, or ones that contain facial piercings (e.g., nose piercings, eyebrow piercings).
12. Photographs must be taken vertically (not sideways/horizontally).
13. Photograph must have a neutral facial expression (e.g., mouth closed, eyes open and clearly visible).
14. Do not use filters commonly used on social media.
15. Photographs should not be digitally altered.
16. Must be comparable to the style of a driver's license or a passport photograph.

Please indicate the approximate date that each photograph was taken and your approximate age.

## Missing persons posters for "Experiment one"

Target one.

Low variability.

High variability.

Target two.

Low variability.

High Variability.

Target three.

Low variability.

High variability.

Target four.

Low Variability.

High Variability.

## Appendix C

Missing persons posters for Experiment two.

Target one:

Low variability.

High variability.

Target two:

Low variability

High variability.

Target three:

Low variability.

High Variability.

## Footnotes

1 In the preregistration, we included the hypothesis that participants who studied high variability photographs would make more overall sightings (hits and false alarms) than participants who studied low variability photographs. In text, we compiled this hypothesis under the signal detection measures. Additionally, hypothesis 1,4, and 6 in the preregistration conflict in terms of predictions about false alarms so we do not include any explicit predictions about false alarms here.

2 In the preregistration, we included the hypothesis that when expectations of encounter are low, high variability photographs will result in more accurate sighting rates (and less inaccurate sighting rates) compared to low variability photographs. However, in a grant application made before the preregistration, we posited the opposite. To reconcile this issue, we decided that either hypothesis was viable and thus offer them as competing hypotheses.

3 Similarity scores between the study and lineup photographs were the same across variability conditions in Experiment 2, except for one of the targets for whom similarity scores were higher between the low variability photographs and lineup photograph than the high variability photographs.
